# Slow wave sleep in naps supports episodic memories in early childhood

**DOI:** 10.1111/desc.13035

**Published:** 2020-09-17

**Authors:** Sanna Lokhandwala, Rebecca M. C. Spencer

**Affiliations:** ^1^ Department of Psychological & Brain Sciences University of Massachusetts Amherst MA USA; ^2^ Developmental Science Program University of Massachusetts Amherst MA USA; ^3^ Neuroscience & Behavior Program University of Massachusetts Amherst MA USA; ^4^ Institute for Applied Life Sciences University of Massachusetts Amherst MA USA

**Keywords:** episodic memory, memory consolidation, napping, preschool education, sleep

## Abstract

Naps have been shown to benefit visuospatial learning in early childhood. This benefit has been associated with sleep spindles during the nap. However, whether young children's naps and their accompanying physiology benefit other forms of declarative learning is unknown. Using a novel storybook task, we found performance in children (N = 22, mean age = 51.23 months) was better following a nap compared to performance following an equivalent interval spent awake. Moreover, performance remained better the following day if a nap followed learning. Change in post‐nap performance was positively associated with the amount of time spent in slow wave sleep during the nap. This suggests that slow wave sleep in naps may support episodic memory consolidation in early childhood. Taken in conjunction with prior work, these results suggest that multiple features of brain physiology during naps may contribute to declarative memory processing in early childhood.

## Graphical abstract

Children remember more of the sequence of events in a storybook if they sleep following learning compared to when they stay awake following learning. This benefit is specifically associated with slow wave sleep in the nap.

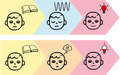

**Research Highlights**

- Midday naps in early childhood benefit episodic learning of a sequence of storybook events.
- Greater time spent in slow wave sleep is associated with better memory following a nap, suggesting that naps actively facilitate the consolidation of episodic memories.
- Taken in conjunction with prior work, these results support a task‐independent benefit of naps on declarative memory consolidation. However, physiology supporting this benefit may be task dependent.

## 1 INTRODUCTION

Early childhood is a critical time for memory development (Shonkoff & Phillips, 2000). Starting around 3 years of age, children begin to display signs of early autobiographical memory (i.e., memory for personal past events; see Bauer, 2015 for review), including the ability to provide verbal accounts of the specific people taking part in past events (Bauer & Saeger Wewerka, 1995). This is also an age at which considerable advances are made in declarative memory (Bauer, 2008; Squire & Zola‐Morgan, 1991). Declarative memory encompasses both semantic memory (i.e., memory for facts) and episodic memory (i.e., memories for specific experiences), and is arguably the form of memory most focused on in formal education and everyday settings. While advancements in declarative memory observed in early childhood have been broadly attributed to brain development (Bauer, 2008; Riggins et al., 2018; see Keresztes, Ngo, Lindenberger, Werkle‐Bergner, & Newcombe, 2018 for review), individual differences in brain development may not completely account for differences in memory development (Riggins, 2014; Sluzenski, Newcombe, & Kovacs, 2006).

Declarative memory consolidation, the off‐line strengthening and stabilization of memories, involves the hippocampus (Eichenbaum, 2000), a region of the brain that undergoes rapid growth during early childhood. By 5 years of age, the brain is approximately 90% of its adult size (Dekaban & Sadowsky, 1978), while hippocampal volume continues to increase through the first decade of life (Blankenship, Redcay, Dougherty, & Riggins, 2017; Uematsu et al., 2012). Newly encoded memory traces are temporarily stored in the hippocampus and are gradually transferred (or transformed; Moscovitch, Cabeza, Winocur, & Nadel, 2016) to more long‐lasting representations in the neocortex (Buzsáki, 1989). Growing evidence suggests that sleep facilitates the transfer of these memories from the hippocampus to the cortex, a process termed sleep‐dependent memory consolidation (Born & Wilhelm, 2012; Maquet, 2001).

Sleep‐dependent memory consolidation has been demonstrated by comparing performance changes over an interval with sleep to performance changes over an equivalent interval awake (Gais & Born, 2004; Peigneux et al., 2004; Wilson, Baran, Pace‐Schott, Ivry, & Spencer, 2012). For instance, young adults who slept after learning a paired‐associates task showed superior memory compared to when they were awake for an equivalent period of time following learning (Payne et al., 2012; Plihal & Born, 1997; Wilson et al., 2012). Studies in children suggest that sleep, including napping, provides a similar benefit on memory at a young age (Desrochers, Kurdziel, & Spencer, 2016; He, Huang, Waxman, & Arunachalam, 2020; Sandoval, Leclerc, & Gómez, 2017; Spanò et al., 2018; Wilhelm, Metzkow‐Mészàros, Knapp, & Born, 2012; Williams & Horst, 2014). In one study, we taught preschool‐aged children (36–67 months) a visuospatial task in which they learned the spatial locations of items on a grid and subsequently recalled their locations (similar to the game “Memory”). Children learned the task in the morning, followed by an immediate recall phase. Their memory for the locations was probed again (delayed recall) in the afternoon, following a nap or an equivalent interval awake (within‐subject, order counterbalanced, conditions separated by 1 week). Children retained what they learned when they napped between immediate and delayed recall but forgot many of the learned items when they stayed awake during this interval. Further, this benefit of the nap on memory formation was maintained when children were tested 24 hrs later (Kurdziel, Duclos, & Spencer, 2013). We concluded that, as seen in adults, declarative memories are consolidated over a nap interval in early childhood.

While numerous behavioral studies support sleep's benefit on memory consolidation, recent studies have begun to address the physiological mechanisms underlying this process. Specifically, both slow wave sleep (SWS) and sleep spindles (bursts of neural activity, 9–16 Hz, characteristic of nREM stage 2 sleep) predict memory changes over sleep in adults, particularly for declarative memories (Baran, Mantua, & Spencer, 2016; Steriade & Amzica, 1998; Wilhelm, Diekelmann, & Born, 2008). The association observed between SWS and memory performance is in line with the active system consolidation hypothesis, which specifies that during SWS, newly encoded memories are repeatedly reactivated in the hippocampus, supporting the transfer of information from the hippocampus to the neocortex (see Rasch & Born, 2013 for review). In one study, cued reactivation of memories during SWS was related to increased activation in the anterior and posterior part of the left hippocampus (Rasch, Büchel, Gais, & Born, 2007). In another study, adults learned to associate spoken words with pictures of scenes or objects. During subsequent nREM sleep, a subset of these spoken words was replayed. Not only was memory superior for these replayed associations compared to items which were not replayed during sleep, but also memory cues during sleep enhanced sleep spindle activity (Cairney, Guttesen, El Marj, & Staresina, 2018). Taken together, these studies suggest that properties of sleep actively play a role in memory consolidation.

While declarative memory has been associated with SWS and spindles in adults, few studies have considered the relevant sleep features which support memory consolidation in early childhood. In a study of visuospatial learning described above, Kurdziel et al. (2013), found a significant positive relation between sleep spindle density and change in memory over the interval with a nap. The relation between sleep spindles and declarative memory consolidation during naps has also been observed in infants (Friedrich, Mölle, Friederici, & Born, 2020) and adolescents (Piosczyk et al., 2013). Mechanistically, sleep spindles and hippocampal ripples (high‐frequency field oscillations; 100–300 Hz) are embedded in slow waves, and these oscillations interact in a fine‐tuned manner to allow the transfer of newly encoded memories (e.g., declarative memories) from the hippocampus to the neocortex (Rasch & Born, 2013; Schabus et al., 2006). Consistent with this, memory consolidation in children has also been associated with slow wave sleep (Wilhelm et al., 2013).

The purpose of the present study was to assess the generalizability of results of Kurdziel et al. (2013). Specifically, from those findings we concluded that sleep benefits declarative learning in early childhood, and such benefits are sleep spindle dependent. However, it may be premature to draw such conclusions without assessing other declarative memory tasks. If such findings are indeed generalizable to other forms of declarative learning, we expect that when probing other declarative memory tasks in young children, (a) memory recall will be greater following a nap compared to recall following an interval awake and (b) the amount of memory protection offered by the nap (i.e., the change in recall from before to after the nap) will significantly correlate with sleep spindle density.

To this end, we used a task which required learning the sequence of events in a storybook to probe episodic memory. This task is both age‐appropriate and has ecological validity. Williams and Horst (2014) used a similar task, probing word learning from the storybook as their outcome variable. They found that napping shortly after learning may be important for word learning compared to staying awake. However, that study used a between‐subjects design, in which children who were habitual nappers were assigned to the nap condition, and non‐habitual nappers were assigned the wake condition. We (Kurdziel et al., 2013) and others (Lam, Mahone, Mason, & Scharf, 2011; Watamura, Donzella, Kertes, & Gunnar, 2004) have hypothesized that children who nap may fundamentally differ from those who no longer nap, with superior brain development and cognitive skills for non‐nappers even when controlling for age. For this reason, we used a within‐subjects design to compare consolidation of memories of event sequences from age‐appropriate storybooks. We hypothesized that naps would benefit memory on this task. However, a reasonable alternative hypothesis that we considered is that naps may have a unique role in visuospatial learning given that such tasks tap the spatial‐detecting place cells of the hippocampus, where neural replay during sleep is most well understood (Ji & Wilson, 2007; Skaggs & McNaughton, 1996).

Our second objective was to determine whether nREM2 sleep spindles play a general role in declarative memory consolidation in early childhood. Based on Kurdziel et al. (2013), we hypothesized that consolidation of episodic storybook learning would likewise be related to sleep spindle density in early childhood naps. However, studies in adults (Backhaus et al., 2007; Baran, Wilson, & Spencer, 2010) and children (Wilhelm et al., 2013) suggest the specific sleep mechanism may be task dependent and that SWS may instead facilitate memory consolidation (Baran et al., 2016; Gais & Born, 2004 for review; Marshall & Born, 2007; Tucker et al., 2006). Thus, we considered the role of both sleep spindles and SWS in episodic memory consolidation in preschool children.

## 2 METHODS

### 2.1 Participants

Participants were 22 children 36–71 months of age (seven females; *M* = 51.23 months, *SD* = 8.95; similar in age to the Kurdziel et al., 2013 study). Children were recruited through the university's Infant and Child Studies Database and through advertising on parent‐directed social media groups. Children were eligible if they had normal or corrected‐to‐normal vision, had no history of diagnosed sleep disorders, were not using any sleep‐affecting or psychotropic medications, had no learning or developmental disabilities, had not recently traveled outside of the local time zone, and were deemed likely to nap in the laboratory. An additional five participants were excluded due to scheduling conflicts (n = 2), inability to comply with study procedures (n = 2), and technical difficulties (n = 1). Additionally, when assessing children's memory performance across the nap and wake condition, performance of one participant was deemed an extreme multivariate outlier (defined as three *SD*s above the average Cook's distance) and was subsequently excluded from all analyses.

### 2.2 Storybook task

**FIGURE 1:**
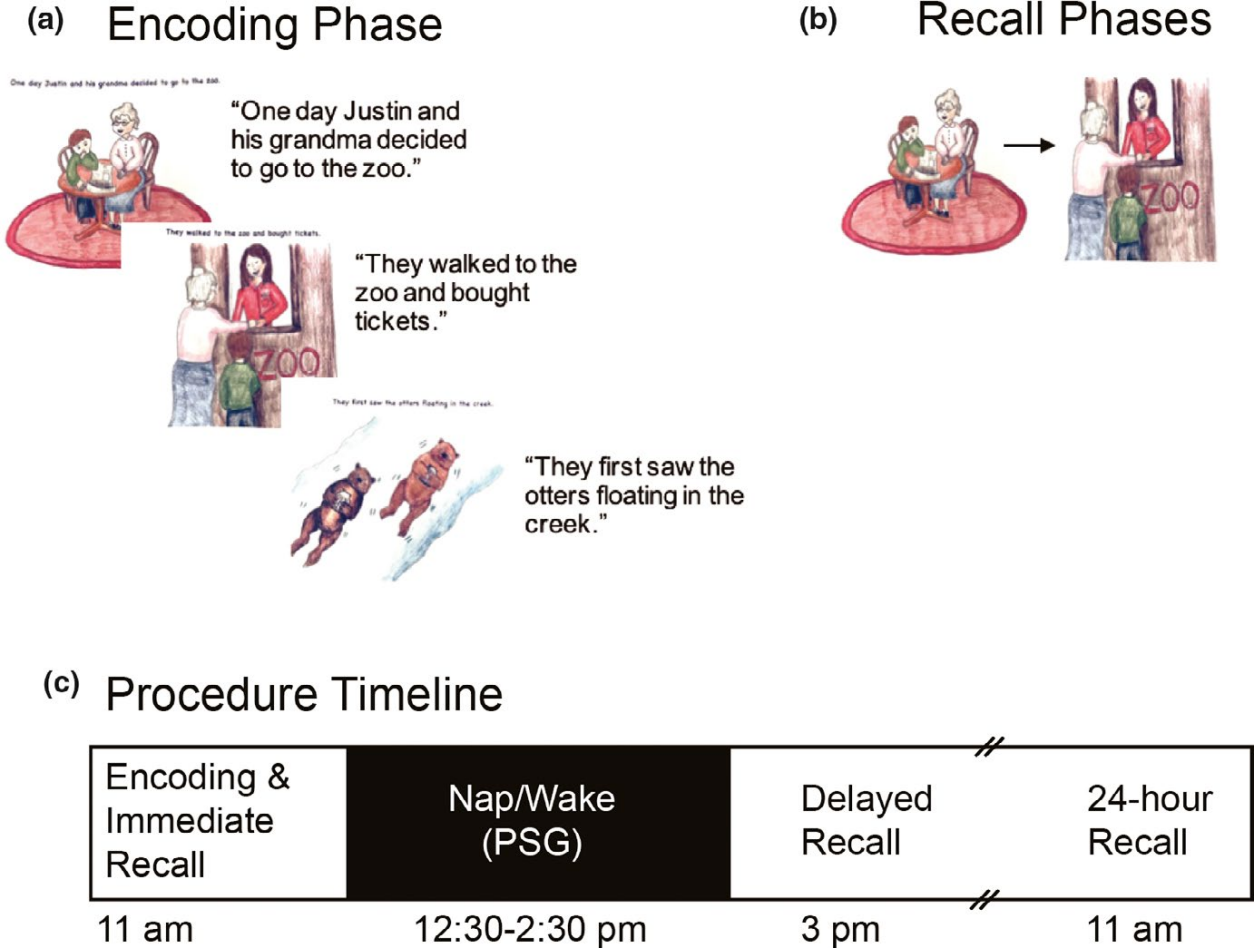
(a) Children were read four 10‐page experimental books. The first page of each book began with a goal sentence with the subsequent nine pages illustrating actions or events. (b) During all three recall time points, children were handed picture cards of the story and asked to put the story back in order. (c) Prior to the midday nap opportunity, children were fitted with a PSG cap, and then, were either nap or wake promoted (the alternate condition taking place approximately 1 week later). Subsequently, delayed recall was tested. They did this once more the following day during 24‐hr recall

Episodic memory was investigated with a story‐based task similar to that of Williams and Horst (2014) and the NIH Toolbox Picture Sequence Memory Test (Bauer et al., 2013). Stimuli consisted of one 5‐page practice book (*Birthday Party)* and four 10‐page experimental books (*Trip to the Zoo*, *Going Camping*, *Making Cookies*, *Playing in the Park)*, all designed in‐house. Books were printed on 18 cm × 11 cm pages. Each book described an age‐appropriate event or activity (e.g., going camping). The first page of each story consisted of a goal sentence complemented by an illustration. The subsequent nine pages depicted a series of events described with a short sentence (Figure 1A). Two books followed a generally logical sequence (e.g., baking cookies), and two books followed a generally arbitrary order of events (e.g., playing in the park) and provided greater difficulty. Encoding consisted of reading two experimental books, one with a logical sequence and one with an arbitrary sequence, with books counterbalanced across nap and wake conditions. To assess memory, 10 picture cards (five for the practice book) capturing the various activities from the story were created for each book.

### 2.3 Sleep measures

Polysomnography (PSG) was acquired during naps using customized 32‐channel PSG electrode caps (EasyCaps; Brain Products GmbH, Germany) with a sampling rate of 500 Hz. The EEG montage included 24 cortical electrodes (Fz, F3, F4, F7, F8, FCz, FC1, FC2, FC5, FC6, C3, C4, CP1, CP2, CP5, CP6, Pz, P3, P4, P7, P8, POz, O1, and O2), two EOG electrodes, and two EMG electrodes. EEG data were recorded relative to ground at FPz and referenced to Cz and the contralateral mastoids (A1 and A2). The EMG leads were referenced to each other.

Children also wore actigraphy watches (Actiwatch Spectrum, Philips Respironics, Bend, OR) on the nondominant wrist continuously for 16 days (fitted approximately 3–4 days prior to the child's first session). The Actiwatch sampled activity at 32 Hz, with a sensitivity of <0.01 g, and activity was stored in 15‐s epochs. Caregivers were instructed to press an event marker to mark the beginning and end of sleep bouts.

### 2.4 Questionnaires

To assess children's general sleep habits, a primary caregiver completed the Child Sleep Habits Questionnaire (CSHQ; Owens, Spirito, & McGuinn, 2000). Primary caregivers also filled out a daily sleep diary for the child, noting sleep onset and offset. An in‐house health and demographics questionnaire completed by parents was used to characterize the participant population.

### 2.5 Procedure

All procedures were approved by the University of Massachusetts Amherst Institutional Review Board. Procedures were based closely on those of Kurdziel et al. (2013; in‐lab portion of that study). Approximately 4 days prior to the first experimental condition, caregivers provided informed consent for their child's participation. Child assent was obtained at each experimental stage. At this time, parents were also given a study packet that included questionnaires and instructions on how to use and care for the Actiwatch. Children were fitted with the Actiwatch. The experimental sessions took place in the sleep lab. Participants were randomly assigned to begin with either a nap or wake condition. The alternate condition took place approximately 1 week later.

For each session, children and their caregivers were scheduled to arrive at the sleep lab approximately 1 hr before the child's typical nap time (typically around 12:00 p.m.). Once comfortable, the child completed the practice phase of the storybook task. In this phase, the child was read the practice book. The experimenter read one page at a time, engaging the child with the story (e.g., “Look at Maddie's birthday cake!”). Next, the child was presented with the picture cards for that story. The child was then asked to place the cards in order as they occurred in the story. Feedback on correct sequencing was provided in this phase.

In the encoding phase, immediately following the practice phase, the experimenter read two stories to the child. The experimenter sat adjacent to the child and read one page at a time, encouraging them to attend to the pictures (no explicit instructions to remember the story sequence were given). Short breaks between the two stories were permitted if the child required them (e.g., going to the bathroom). Prior to the subsequent immediate recall phase (Figure 1B), the experimenter asked the child to recall the story that was read earlier. The child was then presented with picture cards from the stories and asked to put them in the correct order (one story at a time). No feedback was provided.

Following immediate recall, the child was fitted with a PSG cap (approximately 30–50 minutes). In the nap condition, the child was subsequently given a 2‐hr nap opportunity in which sleep was promoted (e.g., via back rubs and lullabies). In the wake condition, the PSG cap ensured wakefulness while they engaged in quiet activities (e.g., puzzles, drawing). Thirty minutes following the nap/wake condition (approximately 3:00 p.m.), children completed the delayed recall phase. The child was again presented with the picture cards and asked to arrange them in order of the storybook without feedback. Subsequently, the caregiver and child left the lab and continued with their day.

In both conditions, the caregiver and child returned to the lab the following day, approximately the same time the stories were read the previous day, for the 24‐hr recall phase. The child was given the story cards once more and asked to put them in the correct order. No feedback was provided.

Following each recall phase, the child self‐reported their sleepiness (Maldonado, Bentley, & Mitchell, 2004) and mood (Stern, Arruda, Hooper, Wolfner, & Morey, 1997) using pictorial Likert scales. Experimenters separately rated sleepiness and mood of the child using the same scales.

## 3 DATA ANALYSIS

### 3.1 Storybook task

Of interest was the change in memory from immediate recall to delayed and 24‐hr recall across the two conditions. Memory performance was measured as the sum of adjacent pairs of events remembered correctly divided by the maximum score. Each correct adjacent pairing received a score of 1, with a maximum score of 9 per storybook (e.g., a reported sequence of 1–3–4–6–5–7–8–2–9–10 would receive a score of 3 as 3–4, 7–8, and 9–10 are correct adjacent pairs). Scores were combined for the two storybooks in each condition for a maximum score of 18. Scores for each testing time point were a proportion of sequences correctly remembered over the two books. Two‐tailed paired‐samples *t* tests were used to compare differences in performance across immediate, delayed, and 24‐hr recall. *p* values below α = 0.05 were considered statistically significant. Analyses were uncorrected for multiple comparisons.

To assess changes in memory following the nap and wake conditions, immediate recall accuracy was subtracted from delayed recall accuracy to create a delayed difference score (i.e., delayed difference score = ((delayed recall − immediate recall)*100)). The difference score was calculated separately for the nap and wake conditions. The 24‐hr difference score reflects the change in recall from immediate recall to the 24‐hr assessment ((24‐hr recall − immediate recall)*100). Finally, the overnight difference score reflects the change in recall from delayed recall to the 24‐hr recall assessment ((24‐hr recall − delayed recall)*100).

One‐way ANOVAs was used to compare changes in recall performance with within‐subject factors Condition (nap vs wake). Pearson's correlations were used to determine relationships between memory performance and sleep physiology.

### 3.2 Polysomnography

Prior to sleep staging, Cz‐referenced PSG recordings were bandpass filtered (0.3–35 Hz EEG, 10–70 Hz EMG), re‐referenced to the contralateral mastoid, then visually scored in 30‐second epochs according to American Academy of Sleep Medicine criteria (Iber, Ancoli‐Israel, Chesson, & Quan, 2007) using Hume (Saletin & Greer, 2015). Sleep staging was verified by a second trained experimenter. Sleep onset was defined as the onset of the first three consecutive epochs of sleep in the recording.

Sleep spindles were autodetected at C3 (C4 in one participant with a bad C3 electrode) using an established algorithm (Ferrarelli et al., 2007), then manually restricted to only spindles identified during nREM2 for further quantification. Briefly, the unfiltered Cz‐referenced EEG signal was re‐referenced to the average mastoid, bandpass‐filtered using a minimum‐order Chebyshev type II filter with passband corner frequencies of 11 and 15 Hz and stopband corner frequencies of 10 and 16 Hz, then converted to an envelope by extracting the peaks of the rectified signal. Spindles were detected when the envelope exceeded an upper threshold of six times the average envelope amplitude, providing a participant‐specific amplitude criterion. Onset and offset times for each detected spindle were defined as the nearest preceding and following points of the envelope falling below a lower threshold of two times the average envelope amplitude. These bandpass filter settings and envelope amplitude thresholds are consistent with those employed by McClain et al., 2016. Peak amplitude and frequency were quantified for each spindle and averaged within subject.

Slow wave activity was characterized as amplitude density in the delta band (0.5–4 Hz) during SWS using the filter‐Hilbert method, as previously described (Jones, Fitzroy, & Spencer, 2019). Raw EEG data from C3 (based on previous literature; Cremone, Kurdziel, Fraticelli‐Torres, McDermott, & Spencer, 2017; C4 in one participant for whom C3 was a bad electrode) were re‐referenced to the averaged mastoid and bandpass filtered from 0.5 to 4 Hz (Butterworth IIR filter, order 2). Regions of the filtered data exceeding ±250 µV were marked as artifact. Delta amplitude envelopes were extracted from the filtered EEG data using the Hilbert transformation, and SWA was calculated as the average per‐second magnitude of the artifact‐free delta amplitude envelope during SWS. PSG analyses were performed in MATLAB using EEGLAB (Delorme & Makeig, 2004), ERPLAB (Lopez‐Calderon & Luck, 2014), and custom in‐house software (PSG power; available upon request).

### 3.3 Actigraphy

Actigraphy data were scored with Actiware software (Philips Respironics) following standard protocols (Acebo et al., 2005). Specifically, participants with less than 3 days of data were excluded from analysis (n = 9) based on previous work using similar criteria (Penpraze et al., 2006). The remaining 13 children's sleep bouts were scored with reference to event markers and sleep diaries (10 of these children had nap data and were used in subsequent analysis). Sleep onset was determined as the first 3 min of uninterrupted sleep, and sleep offset was determined by the last 5 min of continuous sleep (Acebo et al., 2005). Sleep duration was defined as the total minutes of sleep from sleep onset to sleep offset.

## 4 RESULTS

### 4.1 Sample description

Enrolled participants were 81.8% White, 4.5% Black, and 13.6% identified as “other.” Further, 81.8% were not of Hispanic, Latino, or Spanish origin, and 18.2% were Hispanic from Puerto Rican descent. Of the caregivers who completed questionnaires (n = 18), 88.9% were White, 5.6% were Black/African American, and 5.6% were White/American Indian or Alaska Native. For household income, 11.1% reported an annual household income between $20 000 and $40 000/year, 44.4% between $40000 and $70 000/year, 27.8% between $70 000 and $100 000/year, and 16.7% between $100 000 and 150000/year. Of these caregivers, 11.1% reported having some college education, 11.1% having an Associate's Degree, 33.3% having a Bachelor's Degree, and 44% having a Master's Degree or Doctorate.

### 4.2 Memory performance

Children were able to learn the task as demonstrated by >45% accuracy for immediate recall in both the nap and wake conditions. Immediate recall did not differ across conditions (*t*(21) = −.201, *p* = .843; Table 1). Further, there were no significant differences between the storybooks with a logical versus arbitrary order of events at baseline (*t*(21) = 1.232, *p* = .232). Moreover, immediate recall was neither predicted by age (*r* = .133, *p* = .554) nor gender (*t*(20) = .163, *p* = .872).

**TABLE 1:** Memory performance and child behavior ratings during in‐lab visit (standard deviations in parentheses)

	Nap condition	Wake condition	*P* value
Immediate Recall, %	46.23 (21.18)	47.18 (25.19)	.843
Delayed Recall, %	59.68 (23.34)	42.41 (22.43)	.004
24‐hr Recall, %	65.41 (22.73)	46.64 (23.02)	.001
Sleepiness			
Child rated	1.40 (1.05)	2.09 (1.48)	.044
Experimenter rated	1.50 (0.91)	2.14 (1.46)	.059
Mood			
Child rated	1.68 (0.95)	1.95 (1.40)	.248
Experimenter rated	1.82 (0.96)	1.82 (1.33)	>.99

**FIGURE 2:**
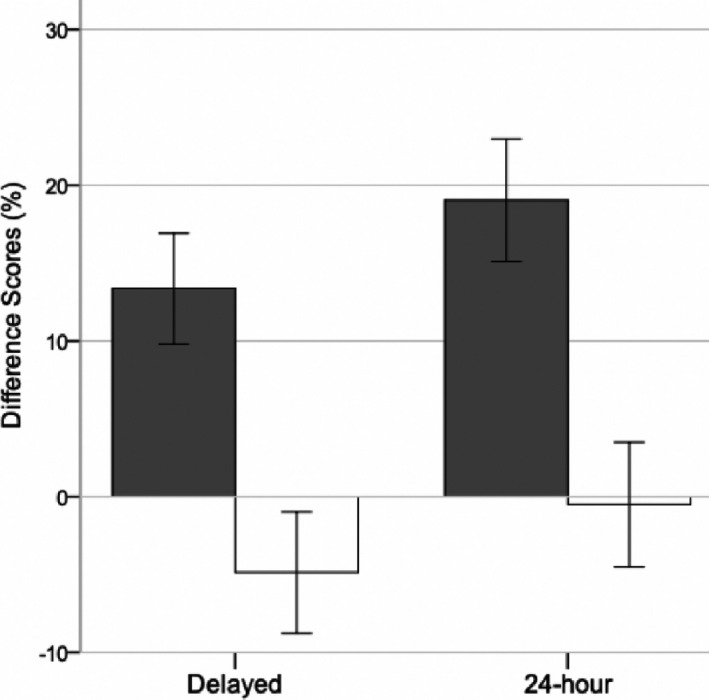
Change in recall accuracy following the nap/wake interval compared to immediate recall (delayed—immediate) was significantly greater in the nap condition (gray bars) relative to the wake condition (white bars; “Delayed”). This nap benefit persisted to the next morning (“24‐hr”). Error bars represent ±1 SE

Similar to the Kurdziel et al. (2013) study, delayed recall accuracy was significantly greater in the nap condition (59.68 ± 4.976) compared to the wake condition (42.41, ±4.782; *t*(21) = 3.272, *p* = .004; Table 1). To control for baseline memory performance, we compared the delayed difference scores across conditions and found a main effect of Condition (*F*(1,21) = 9.258, *p* = .006; Figure 2A).

We considered whether better recall following the nap reflected impairments in the wake condition as opposed to benefits of the nap condition. In other words, children's behavior following nap deprivation (inattentive, emotionally dysregulated) may have impaired performance. There were no differences in child‐ or experimenter‐reported mood following the nap and wake intervals (*p*s > .248). However, child‐rated sleepiness was greater following an interval spent awake (*t*(21) = −2.143, *p = *.044) and experimenter‐rated sleepiness of the child was marginally significant (*t*(21) = −1.993, *p* = .059).

Considering this, we examined whether sleepiness accounted for differences in performance following nap and wake intervals. Both child‐ and experimenter‐rated sleepiness was associated with change in performance following wake (*p*s = .008), but this was not the case following the nap (*p*s > .776). When the ANOVA comparing delayed difference scores across conditions was repeated with sleepiness as covariate, the main effect of condition remained significant (*F*(1,20) = 4.550, *p* = .046).

Moreover, if sleepiness accounted for acute differences, it would be expected that these differences would dissipate the next day, following unrestricted overnight sleep. Like the Kurdziel et al. (2013) study, children continued to perform significantly better when they had napped following learning the prior day compared to when they had not napped (24‐hr recall accuracy: *t*(21) = 3.947, *p* = .001; Table 1). This next‐day nap benefit remained when controlling for immediate baseline performance (*F*(1,21) = 14.086, *p* = .001; Figure 2B). This suggests that the benefit of the nap compared to the wake condition could not be attributed to short‐term behavioral differences across the conditions.

Given that overnight sleep has also been shown to support memory consolidation, we considered whether memory consolidation took place over the overnight interval. There was improvement in mean accuracies from delayed recall to 24‐hr recall (Table 1), which may suggest that memories were consolidated with overnight sleep regardless of condition. However, the overnight difference scores (24‐hr‐delayed recall), collapsed across conditions, were not significantly greater than 0 (*t*(21) = 1.549, *p* = .136), indicating that there was no consistent improvement with overnight sleep. Moreover, the overnight difference scores did not differ across sleep and wake conditions (*F*(1,21) = .105, *p* = .749).

In an exploratory analysis, we considered whether errors made at 24‐hr recall were the same as those made at delayed recall or novel errors. We compared errors made at delayed and 24‐hr recall. Sequencing errors were then inspected for persistent errors (incorrectly matched pairs at both delayed and 24‐hr recall) versus novel errors. The percent of novel errors at 24‐hr recall was calculated as the number of mistakes novel to 24‐hr recall, divided by the total number of errors made at 24‐hr recall. Of those participants making errors at 24‐hr recall (n = 18), 65% of errors were novel in the nap condition. This did not differ significantly from the wake condition, in which 69% of errors were novel at 24‐hr recall (*t*(17) = −1.191, *p* = .250). Further, there was a significant correlation between delayed and 24‐hr recall performance for both the nap and wake condition (*p*s < .005). This suggests that the benefit of sleep after learning persisted after overnight sleep.

### 4.3 Relations between memory and nap physiology

Actigraphy was used to assess habitual sleep (Table 2). On average, children had 3.28 naps/week. Caregivers reported 4.31 naps/week on average (from CSHQ responses). This difference reflects that more children had CSHQ data than actigraphy data. Consistent with definitions in Kurdziel et al. (2013), those that had <2 naps/week were considered non‐habitual nappers, and those with ≥5 naps/week were considered habitual nappers. Three children were non‐habitual nappers (<2 naps/week), three children were habitual nappers (≥5 naps/week), and the remaining children (N = 4) were intermediate nappers (2–4 naps/week). Given the low number of habitual and non‐habitual nappers, we did not conduct additional comparisons of these groups.

**TABLE 2:** Descriptive variables for actigraphy and polysomnography‐recorded naps (standard deviation in parentheses)

	Actigraphy (n = 10)	Polysomnography (n = 22)
Naps/week	3.28 (1.78)	—
Total sleep time (nap length in min)	89.85 (28.53)	94.18 (13.22) range: 73–114 min
Time in bed (min)	97.22 (31.84)	121.55 (8.63)
Sleep onset latency (min)	4.0 (4.8)	14.93 (8.75)
nREM1, %	—	8.59 (5.76)
nREM2, %	—	35.22 (11.92)
SWS, %	—	55.82 (14.17)
REM, %	—	0.37 (1.17)
Spindle density (spindles per minute of nREM2)	—	0.72 (0.38)
Spindle counts (nREM2)	—	23.27 (11.78) range: 1–44 spindles
Spindle frequency (Hz)	—	12.91 (0.32)
Spindle amplitude (µV)	—	27.99 (8.02)

Sleep stage% calculated as ratio from total sleep time.

Abbreviations: REM, rapid eye movement; SWS, slow wave sleep.

Average actigraphy‐recorded nap length was 89.85 min (SD = 28.53 min). Average length of PSG‐recorded naps in the laboratory was 94.18 min (SD = 13.22 min). Naps included little (n = 3, 1–10 min) to no (n = 19) REM sleep. Consistent with prior work, naps were largely comprised of nREM stage 2 sleep and SWS (Cremone et al., 2017; Kurdziel et al., 2013; Table 2).

**FIGURE 3:**
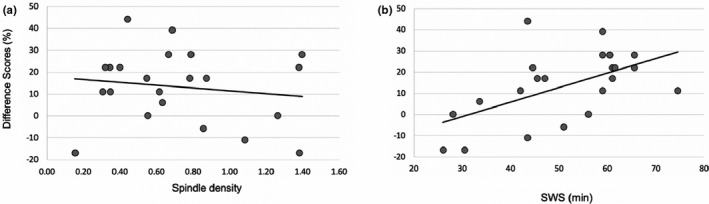
(a) Associations between change in recall accuracy (delay—immediate) and sleep spindle density (number of spindles per minute of non‐REM stage 2; *r* = −.145) and (b) time spent in SWS (*r* = .551)

We tested the hypothesis that the sleep‐spindle‐dependent declarative memory benefit observed in our prior work (Kurdziel et al., 2013) would generalize to the present task. However, spindle density was not associated with the delayed difference score from the nap condition (*r* = −.145, *p* = .520; Figure 3A) nor was nap spindle density associated with the 24‐hr difference score (*r* = .265, *p* = .233). Next, we tested the alternative hypothesis that SWS supported the change in memory over the nap interval. We found a positive significant correlation between time spent in SWS and the nap delayed difference score (*r* = .551, *p* = .008; Figure 3B). The correlation between nap SWS and the 24‐hr difference score was not significant (*r* = .008, *p* = .971). The correlation between slow wave activity (SWA; delta activity in slow wave sleep) and the nap delayed difference score was also not significant (*r* = .206, *p* = .369).

## 5 DISCUSSION

Our previous work (Kurdziel et al., 2013) suggests that declarative memory consolidation over a nap is a function of sleep spindles. Here, we investigated whether this spindle‐dependent nap benefit is generalizable to other declarative memory tasks or, rather, is task specific. Our data indicate that episodic memory is benefited following a nap. Furthermore, this nap benefit extends to the next day, as children performed better approximately 24 hrs after learning when they had napped the day before compared to when they had stayed awake during the nap interval. Our findings thus suggest that sleep close to learning has lasting effects on memory retention for young children, and that overnight sleep cannot undo the adverse consequences of nap deprivation on memory consolidation.

Further, in examining nap physiology, we found that time spent in SWS during the nap may underlie nap‐dependent changes in performance for episodic memory. This is in contrast to our previous work, which found a relationship between nap‐dependent consolidation of visuospatial learning and sleep spindle density. This inconsistent association between memory performance and sleep physiology is reflective of developmental work thus far. For example, work in older children (8–11 years) found that explicit sequence knowledge was associated with higher SWA (Wilhelm et al., 2013), and, similarly, children's (8–12 years) recall of word pairs was positively associated with SWS (Wang, Weber, Zinke, Noack, & Born, 2017). In contrast, work in infants (9–16 months) has found spindle activity to be associated with generalization (Friedrich, Wilhelm, Born, & Friederici, 2015) as well as episodic memory (14–17 months; Friedrich et al., 2020).

We consider two alternatives. One, this association between SWS and memory performance in contrast to Kurdziel's results may indicate that the physiological mechanism underlying declarative memory consolidation is task dependent. That is, tasks that involve remembering spatial context may be more reliant on sleep spindles, while tasks that involve episodic sequences may be more reliant on SWS. Alternatively, it is possible that findings implicating sleep spindles and the present result implicating SWS may actually be pointing to the same mechanism. Specifically, sleep spindles are embedded in slow waves, and it may be this embedded mechanism which supports the consolidation of various forms of declarative memories. It is unclear why different relations emerge for the two studies. However, studies in adults likewise point to the ripple‐spindle‐slow‐oscillation mechanism, even though rarely does more than one association reach significance. This is an important area for future research.

Interestingly, there is not much forgetting over wake (Table 1) on this task. Rather, memories appear protected over wake and enhanced by sleep. This is likely an artifact related to the fact that children are being tested on identical recall probes. That is, children are asked to put the same stories together at three different time points. Interestingly, the majority of the sequencing errors at 24‐hr recall were different from the errors made at delayed recall. Still, there is a significant association between delayed and 24‐hr recall performance. Thus, it is difficult to rule out a practice effect from re‐encoding the stories at delayed recall. Further, the change in performance with overnight sleep (24‐hr recall—delayed recall) was not statistically significant. The lack of difference between overnight performance in the nap and wake condition makes it difficult to suggest that the nap and overnight bouts are interacting to create a benefit that extends beyond practice effects. However, if this was merely a practice effect, we would expect there to be a steady increase at each time point irrespective of napping or being nap deprived. This underlines the adverse consequence of missing out on the midday nap on memory consolidation.

Prior work found children who nap regularly experienced the greatest loss in learning when deprived of the nap compared to non‐habitual nappers (Kurdziel et al., 2013). This finding has been thought to reflect differences in brain maturation, with non‐habitual nappers having more mature memory networks and thus a decreased need for frequent consolidation. We did not have a sufficient number of non‐habitual nappers (<2 nap/week) enrolled in the study to consider the role of nap habituality in our findings. Actigraphy‐estimated nap habituality suggest that the majority of children were also not habitual nappers (≥ 5 naps/week). Thus, the current sample did not allow for disentangling whether change in performance following nap/wake is independent of napping status.

Another limitation of the study is the absence of overnight physiology. Without the overnight component, it is difficult to disentangle whether a midday nap affects overnight physiology. For example, it may be that when a child is kept awake during the nap opportunity, they may have more SWS overnight. Thus, it cannot be discerned whether greater 24‐hr SWS leads to better performance across the two sleep bouts. However, this is unlikely as it cannot explain our finding that greater SWS during children's naps predicted better memory performance immediately following the nap.

Finally, it is important to note that the present task, like the visuospatial task, may not be fully representative of all declarative memory tasks, and some task‐specificity may still exist. While we characterize the sequential ordering of a storybook as emblematic of episodic memory, it may be beneficial to consider a more traditional episodic memory task, such as a free recall task (i.e., a list of to‐be‐remembered items) where the order in which items are remembered is unimportant. Such a task may allow for memories to be “internally cued” by a particular cognitive process (e.g., episodic cognition) versus “externally cued” by evidence in the environment (e.g., picture cards from a story).

Together, these data suggest that naps support early education goals for preschool‐age children. Given evidence that sleep‐dependent consolidation is important for children's learning, compromising nap time in the classroom may hinder children's ability to effectively form memories. Findings from the current study not only suggest that sleep close to learning is beneficial to episodic learning, but also that SWS present in these naps may facilitate the consolidation of such memories. Thus, preserving the nap opportunity should be a valued part of the preschool curriculum.
